# Practical Departments

**Published:** 1903-02-28

**Authors:** 


### PRACTICAL DEPARTMENTS.

### NOTTINGHAMSHIRE SANATORIUM FOR CONSUMPTIVES.

Referring to our criticisms of the Nottinghamshire Sanatorium in our issue of February 14th, the constructors of the building, the Portable Building Company of Fleetwood, point out that the superstructure, omitting the foundations, cost at the rate of £85 per bed, and that this should be borne in mind in considering the total expenditure of £220 per bed, which we observed to be a somewhat high figure. The company observe that in another case the foundations and superstructure together worked out at £90 per bed, which proves that the excellent foundations at Nottingham are, if not extravagant, certainly very expensive.

